# High-Dose Busulfan and Thiotepa Followed by Autologous Stem Cell Transplantation for Refractory Choriocarcinoma with Central Nervous System Metastases: A Case Report

**DOI:** 10.1159/cro/abzag042

**Published:** 2026-09-08

**Authors:** Julian Rapp, Reinhard Marks, Jesus Duque Afonso, Jürgen Finke, Michael Lübbert, Lucas Becker, Urs Würtemberger, Jamal Akkad, Uwe Martens, Cornelius F. Waller, Tim Struessmann

**Affiliations:** a Department of Hematology, Oncology and Stem Cell Transplantation, Medical Center - University of Freiburg, Faculty of Medicine, University of Freiburg, Freiburg, Germany; b Department of Neuroradiology, Medical Center - Faculty of Medicine, University of Freiburg, Freiburg, Germany; c SLK-Kliniken GmbH, Cancer Center Heilbronn-Franken, Klinik für Innere Medizin III, Heilbronn, Germany

**Keywords:** Chemotherapy, Choriocarcinoma, Metastases, Case report

## Abstract

**Introduction:**

Choriocarcinoma is particularly challenging to treat when it has metastasized to the central nervous system. Although metastatic choriocarcinoma generally responds well to therapy, brain metastases are associated with markedly poorer survival and can lead to severe neurological complications. One of the main challenges is that standard chemotherapy regimens, such as high-dose platinum derivatives and etoposide, do not effectively cross the blood-brain barrier, preventing adequate control in the central nervous system. For many, often relatively young patients, treatment options become severely limited when radiotherapy is contraindicated or has already been administered and surgery is not feasible.

**Case Presentation:**

In this study, we describe the successful repurposing of a high-dose chemotherapy regimen consisting of busulfan and thiotepa in a patient with brain metastatic choriocarcinoma unresponsive to carboplatin and etoposide. These agents, widely used in central nervous system lymphoma, are well known for their ability to penetrate the blood-brain barrier. Treatment with this regimen resulted in stable disease and, most importantly, restored a very good quality of life for the patient.

**Conclusion:**

This case suggests a practical and novel therapeutic approach for managing choriocarcinoma with brain metastases, particularly in patients who have already received radiotherapy and are not candidates for further irradiation or surgery.

## Introduction

Choriocarcinoma is a highly aggressive germ cell tumor characterized by rapid hematogenous spread to distant organs. While pulmonary and hepatic metastases are common, central nervous system (CNS) metastases occur in less than 10% of choriocarcinoma patients, making them rare but associated with serious complications and poor prognosis [1]. Treatment of choriocarcinoma with CNS metastases remains challenging. Treatment options in such cases may include radiation, neurosurgery, and chemotherapy, although most approaches are multimodal [2]. Determining the optimal treatment is particularly challenging when radiation or surgery is not feasible due to the anatomical location of the lesions, prior or ongoing interventions, or the need for chemotherapeutic agents to penetrate the blood-brain barrier.

## Case Report

We herein present the case of a 51-year-old male patient who initially attended the emergency department in January 2023 with chest pain, jaundice, and a general decline in performance status. Computed tomography (CT) revealed multiple suspicious lymph nodes in the thoracic, retroperitoneal, and para-aortic regions. Laboratory tests showed elevated peripheral blood β-hCG and lactate dehydrogenase levels of 13,074 mIU/mL (Fig. 1a) and 509 U/L, respectively, while the AFP level was below 2 µg/L. A surgical lung biopsy was performed and revealed a neoplastic cell population positive for pancytokeratin and β-hCG. The tumor cells were negative for CD31, CD34, WT1, D2-40, and PLAP, and showed a high Ki-67 proliferation index. Immunohistochemical findings further confirmed the results and the diagnosis of a pure choriocarcinoma. The primary tumor site remained unknown. The disease was classified as stage IIIB according to the American Joint Committee on Cancer and Union for International Cancer Control staging systems (cTx cN3 pM1a S2), with an intermediate-risk classification according to the International Germ Cell Cancer Collaborative Group. A formal urological assessment, including testicular ultrasonography, was not performed because of the patient’s extensive pulmonary disease and the urgent need to initiate systemic therapy. The complete timeline of the patient’s diagnostic work-up and treatment course is presented in Table 1. Plan for initial treatment consisted of four cycles of polychemotherapy with PEI (cisplatin 20 mg/m^2^, etoposide 100 mg/m^2^ and ifosfamide 1,200 mg/m^2^ d1-5, every 3 weeks) [3]. PEI was selected to avoid the use of bleomycin because of the extensive pulmonary disease and the associated risk of further pulmonary complications. During the first cycle of PEI, the patient required admission to intensive care due to hypoxic respiratory failure caused by severe tumor lysis syndrome and pneumonia (Table 1). Veno-venous extracorporeal membrane oxygenation (VV-ECMO) was required. The intensive care course was further complicated by an iatrogenic tension pneumothorax and pulmonary aspergillosis, based on Aspergillus PCR positivity in bronchoalveolar lavage fluid, which was treated with voriconazole. Following microbiological and respiratory improvement, VV-ECMO was successfully discontinued. Due to the complications, a modified chemotherapy protocol with reduced toxicity was administered for the next two cycles (carboplatin 6 AUC d1, etoposide 120 mg/m^2^ d1-3, every 3 weeks) and not continued with PEI (Table 1). However, the patient experienced a recurrence of hypoxic respiratory failure due to tumor lysis syndrome and pneumonia, requiring a further two cycles of VV-ECMO. No pathogen was identified on either occasion. However, because nosocomial pneumonia was suspected, empirical treatment with piperacillin/tazobactam, linezolid, and caspofungin was initiated, resulting in an improvement in respiratory function. During the intensive care stay, prolonged mechanical ventilation necessitated tracheostomy. VV-ECMO support was successfully weaned on each occasion following antimicrobial therapy and subsequent respiratory improvement (Table 1).

**Fig. 1.**
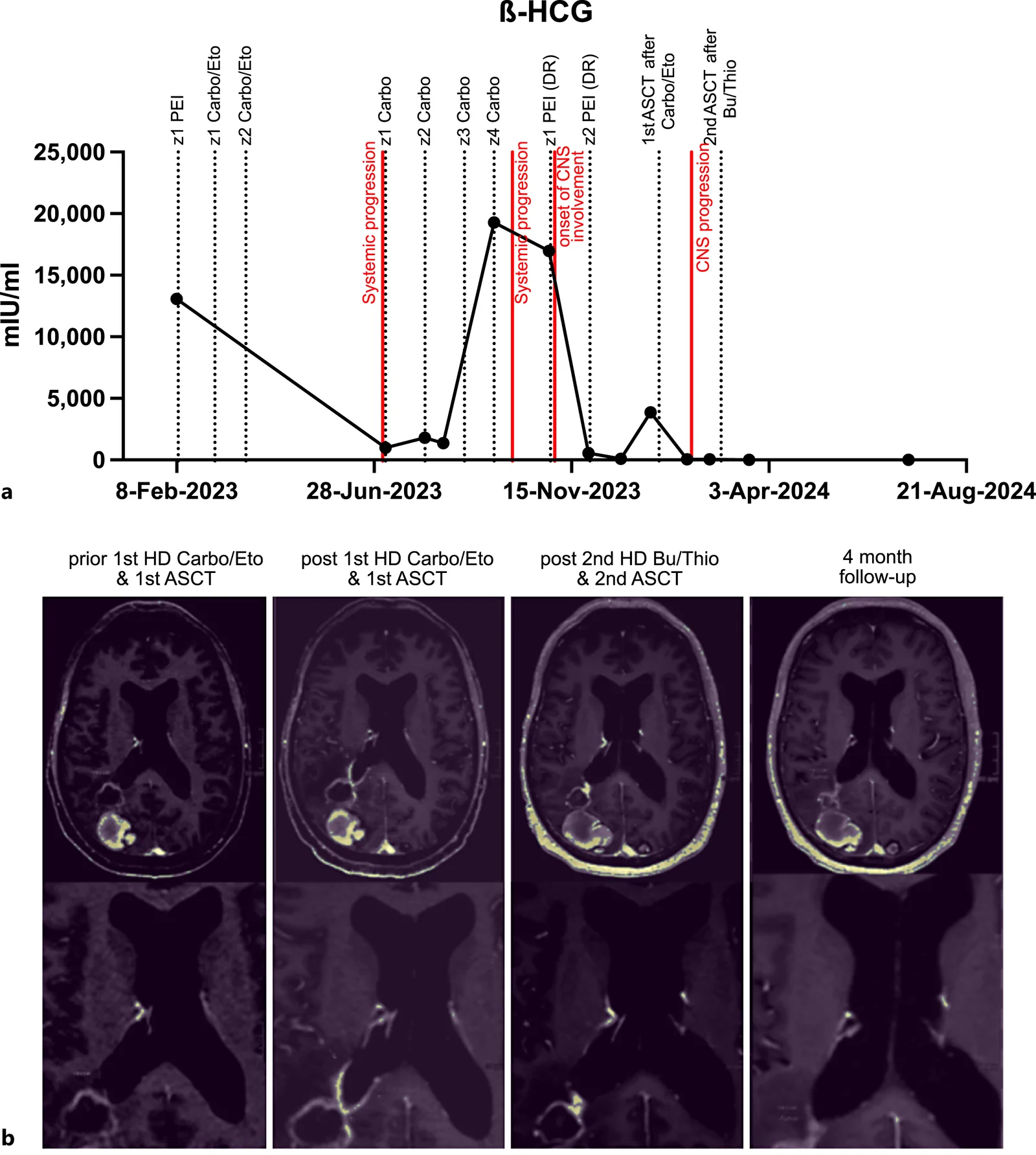
β-HCG levels and CNS progression by MRI during treatment of the patient. **a** Line diagram illustrating the patient’s β-HCG levels in mIU/mL, with the administration of respective treatments and autologous stem cell transplant (ASCT) also indicated. **b** MRI scans of the patient were performed at multiple time points: prior to the first high-dose chemotherapy with carboplatin and etoposide and ASCT; after the first session, which showed progressive disease; 1 month after the second high-dose chemotherapy under busulfan/thiotepa; and at the 4-month follow-up.

**Table 1.** Diagnostic evaluation and treatment course of the patient

Date	Treatment/investigation	Clinical context/rationale
16 Jan 23	CT of the chest and abdomen showed centrally necrotic masses in the retroperitoneal, left iliac, and pulmonary regions	Initial radiological assessment
19 Jan 23	Partial lung resection with histopathological confirmation of choriocarcinoma	Diagnostic tissue sampling
09 Feb 23	Cycle 1 PEI: cisplatin 20 mg/m^2^, etoposide 100 mg/m^2^, and ifosfamide 1,200 mg/m^2^ on days 1–5. Only days 1–3 were administered	PEI was selected as first-line treatment instead of PEB to avoid bleomycin because of the patient’s extensive pulmonary disease
28 Mar 23	Acute respiratory distress syndrome requiring VV-ECMO	​
07 Mar 23	Cycle 1 carboplatin/etoposide: carboplatin AUC 6 on day 1 and etoposide 120 mg/m^2^ on days 1–3	Treatment was changed because of severe tumor lysis syndrome and ARDS during PEI.
21 Mar 23	Recurrent ARDS requiring VV-ECMO	​
29 Mar 23	Cycle 2 carboplatin/etoposide: carboplatin AUC 6 on day 1 and etoposide 120 mg/m^2^ on days 1–3	​
02 Apr 23	Recurrent ARDS requiring VV-ECMO	​
06 Jul 23	Cycle 1 carboplatin monotherapy: carboplatin AUC 5 on day 1	Treatment was de-escalated because of recurrent severe tumor lysis syndrome and ARDS during carboplatin/etoposide
03 Aug 23	Cycle 2 carboplatin monotherapy: carboplatin AUC 5 on day 1	​
31 Aug 23	Cycle 3 carboplatin monotherapy: carboplatin AUC 5 on day 1	​
21 Sep 23	Cycle 4 carboplatin monotherapy: carboplatin AUC 5 on day 1	​
04 Oct 23	CT of the neck, chest, abdomen, and pelvis showed progressive disease	Radiological disease progression
31 Oct 23	Cycle 1 dose-reduced PEI: cisplatin 20 mg/m^2^, ifosfamide 1,200 mg/m^2^, and etoposide 50 mg/m^2^ on days 1–3; days 4 and 5 were omitted	PEI was reintroduced because of progressive disease, with dose reduction due to the previous severe tumor lysis syndrome and ARDS
03 Nov 23	Brain MRI demonstrated the initial diagnosis of CNS metastases	​
04 Nov 23	Start of whole-brain radiotherapy: total dose 30 Gy in 10 fractions of 3 Gy	​
28 Nov 23	Cycle 2 dose-reduced PEI: cisplatin 20 mg/m^2^, ifosfamide 1,200 mg/m^2^, and etoposide 50 mg/m^2^ on days 1–3; days 4 and 5 were omitted	​
21 Dec 23	Peripheral blood stem cell collection: 10.71 × 10^6 CD34+ cells/kg collected	​
10 Jan 24	CT of the chest and abdomen and brain MRI showed progressive disease	​
12 Jan 24	First high-dose chemotherapy: etoposide 500 mg/m^2^ and carboplatin AUC 6 on days 1–3	​
16 Jan 24	First autologous stem cell transplantation with 3.57 × 10^6 CD34+ cells/kg	​
08 Feb 24	Brain MRI showed further CNS progression	​
21 Feb 24	Second high-dose chemotherapy: intravenous busulfan 3.2 mg/kg on days 1–2 and intravenous thiotepa 5 mg/kg on days 3–4	Individualized treatment attempt because of refractory CNS disease after standard systemic treatment and whole-brain radiotherapy, with no feasible surgical or additional radiotherapy options
29 Feb 24	Second autologous stem cell transplantation with 3.57 × 10^6 CD34+ cells/kg	​
17 Apr 24	Stable disease on CT and brain MRI.	​
11 Jul 24	Stable disease on CT and brain MRI.	​
01 Oct 24	Stable disease on CT and brain MRI.	​
13 Jan 25	Stable disease on CT and brain MRI.	​
Jul 2025	Stable disease on CT and brain MRI.	​
Sep 2025	Stable disease on CT and brain MRI.	​
Jan 2026	Stable disease on CT and brain MRI.	​
Apr 2026	Stable disease on CT and brain MRI.	​

Four cycles of carboplatin monotherapy (5 AUC d1) were then administered [4] to reduce treatment-related toxicity compared with carboplatin/etoposide (Table 1). Staging in October 2023 showed progression of pulmonary metastases accompanied by rising β-HCG levels of 19,273 mIU/mL (Fig. 1a). Salvage therapy was initiated with two cycles of reduced-dose PEI (cisplatin 20 mg/m^2^, ifosfamide 1,200 mg/m^2^ and etoposide 50 mg/m^2^ d1-3, removal of d4-5). Although the initial PEI course had been complicated by severe tumor lysis syndrome and ARDS requiring VV-ECMO, these complications were considered to be related primarily to the high tumor burden and rapid treatment response rather than unequivocal chemotherapy resistance. PEI was, therefore, resumed as salvage therapy at a reduced dose and shortened schedule, with omission of days 4 and 5, to balance the potential antitumor benefit against the substantial risk of recurrent toxicity (Table 1). During the first cycle of PEI, the patient reported visual disturbances followed by sudden loss of vision. No prior CNS involvement had been documented, and the patient had not exhibited any neurological abnormalities before. Magnetic resonance imaging (MRI) revealed multiple new intracerebral metastases involving the occipital lobes and cerebellum. The largest lesions measured 20 × 20 × 21 mm in the right occipital lobe and 23 × 28 × 24 mm in the left occipital lobe, with an additional caudal left occipital lesion measuring 9 × 8 × 12 mm. Further lesions were identified in the right periventricular white matter adjacent to the lateral ventricle (5 mm), the right cerebellar hemisphere (16 × 12 × 12 mm), and the left cerebellar hemisphere with contact to the tentorium (25 × 23 × 17 mm). Additional small non-enhancing lesions were present in the right occipital white matter and the right frontal base (Table 1). The larger lesions were associated with marked perifocal edema, compression of the lateral ventricular horns, and a 5-mm rightward midline shift. One occipital lesion showed central necrosis and hemorrhage, accounting for the patient’s visual symptoms. Cranial whole-brain radiotherapy consisting of 30 Gy in 10 fractions parallel with the first cycle of PEI and dexamethasone therapy restored the patient's vision. Radiological staging in January 2024, prior to planned high-dose chemotherapy, showed disease progression in both lung and CNS. High-dose chemotherapy (etoposide 500 mg/m^2^ and carboplatin 6 AUC d1-3) was started and autologous stem cell transplantation was performed on day 5 [5]. A total of 3.57 × 10^6 CD34+ cells/kg were infused on day 0 (Table 1). The patient engrafted successfully without major complications by the end of January. However, due to neurological symptoms such as cognitive functional limitations, a central MRI scan was performed at the beginning of February and showed further progression of the CNS metastases (Fig. 1b), though β-HCG levels remained low (Fig. 1a). In view of the CNS disease progression and the lack of reasonable surgical or radiotherapy options, an individualized treatment plan with CNS-targeted high-dose chemotherapy (busulfan 3.2 mg/kg d1-2 intravenous administration, thiotepa 5 mg/kg d3-4 intravenous administration, stem cell support on day 9) was implemented. High-dose busulfan and thiotepa-based chemotherapy is well established in the therapy of CNS lymphoma patients due to its ability to cross the blood-brain barrier, resulting in effective concentrations in the brain parenchyma [6]. In the absence of established dosing recommendations for solid tumors, busulfan and thiotepa were administered according to the dosing schedule used in CNS lymphoma. Supportive treatment included seizure prophylaxis with levetiracetam (500 mg twice daily, days −8 to −5) and bromazepam (3 mg once daily at bedtime, days −8 to −4). Continuous antifungal prophylaxis with fluconazole 200 mg once daily was administered until engraftment. Antiviral prophylaxis with aciclovir 400 mg was initiated after transplantation. Prophylaxis against hepatic sinusoidal obstruction syndrome consisted of ursodeoxycholic acid (Ursofalk) 250 mg twice daily. A total of 3.57 × 10^6 CD34+ cells/kg were infused on day 0 (Table 1). The second transplantation was performed 6 weeks and 2 days after the first transplantation. Grade 3 mucositis was the main treatment-related complication and required temporary continuous intravenous piritramide administration until engraftment. In addition, the patient developed a grade 3 infection requiring intravenous antibiotic therapy until engraftment. The patient remained cardiorespiratorily stable throughout treatment. A mild transient increase in alanine aminotransferase was observed, from 306 U/L at start of treatment to a peak level of 354 U/L, corresponding to grade 1 according to CTCAE criteria. No neurological toxicity can be reported. Platelet engraftment occurred on day +8 and neutrophil engraftment on day +10 after autologous stem cell transplantation.

After high-dose chemotherapy with busulfan and thiotepa, the patient was discharged in March 2024. Staging in April and July showed stable disease in the thoracoabdominal region and especially in the CNS (Fig. 1b). β-HCG levels remained as low as <1 mIU/mL (Fig. 1a). The patient is being closely monitored with follow-up visits, including MRI and CT imaging. At the most recent follow-up in April 2026, the serum β-hCG level was <1 mIU/mL. CT and MRI findings continued to show stable disease. The patient reported being in good overall health and resumed his previous occupation. This case report was prepared in accordance with the CARE guidelines. The completed CARE checklist is available as supplementary material.

## Discussion

This case report highlights the challenges of treating CNS metastases from choriocarcinoma, particularly when surgery or further radiotherapy is not feasible or when the disease is refractory to these treatment modalities. High-dose chemotherapy for choriocarcinoma, particularly carboplatin, penetrates the blood-brain barrier by only 3–4% [7], making its efficacy for CNS lesions questionable. A palliative regimen with paclitaxel, oxaliplatin, and gemcitabine, standard after progression post–stem cell transplantation [8], was discussed but not chosen as the patient’s β-HCG had normalized, CNS progression was the main concern, and a curative approach was desired. After achieving stable disease with busulfan/thiotepa-based high-dose chemotherapy [6], choriocarcinoma appeared to partially respond to these drugs. To our knowledge, this is the first report of such a treatment. In non-CNS relapsed choriocarcinoma, multiple high-dose carboplatin/etoposide cycles with autologous stem cell transplantation can extend remission [5]. However, repeating high-dose busulfan/thiotepa for CNS control appears unfeasible due to the high anticipated toxicity of repeated cycles.

High-dose chemotherapy with carboplatin and etoposide, followed by autologous stem cell transplantation, yields a median overall survival of about 5 years [5]. In comparison, salvage regimens with paclitaxel, oxaliplatin, and gemcitabine achieve only 6–9 months [8]. However, patients with CNS metastases account for fewer than 10% of these cohorts and have a substantially poorer prognosis, with a median survival of only 8 months following the onset of CNS lesions. [2]. For such cases, busulfan and thiotepa may offer a promising alternative for managing complex CNS progression.

## Statement of Ethics

This study was performed in accordance with the Declaration of Helsinki. Ethics approval was not required for this study in accordance with local or national guidelines as the therapy was administered as an individual therapeutic attempt following a tumor board decision. The patient provided informed consent. This retrospective review of patient data did not require ethical approval in accordance with local guidelines. Written informed consent was obtained from the individual for publication of the details of their medical case and any accompanying images.

## Conflict of Interest Statement

The authors have no conflicts of interest to declare.

## Funding Source

This study was supported by the Open Access Publication Fund of the University of Freiburg.

## Author Contributions

Conceptualization, data curation, formal analysis, funding acquisition, investigation, visualization, and writing – original draft: J.R. and T.S. Methodology: J.R., R.M., J.D., J.F., M.L., L.B., U.W., J.A., U.M., C.F.W., and T.S. Project administration and resources: R.M., J.D., J.F., M.L., L.B., U.W., J.A., U.M., and C.F.W. Software: L.B. and U.W. Supervision: R.M., J.D., J.F., and M.L. Validation: L.B., J.A., U.M., C.F.W., and T.S. Writing – review and editing: J.R., R.M., J.D., J.F., M.L., L.B., J.A., U.M., C.F.W., and T.S.

## Data Availability

All data generated or analyzed during this study are included in this article. Further inquiries can be directed to the corresponding author.
